# Cytoskeleton Rearrangements Modulate TRPC6 Channel Activity in Podocytes

**DOI:** 10.3390/ijms22094396

**Published:** 2021-04-22

**Authors:** Alexey Shalygin, Leonid S. Shuyskiy, Ruslan Bohovyk, Oleg Palygin, Alexander Staruschenko, Elena Kaznacheyeva

**Affiliations:** 1 Institute of Cytology, Russian Academy of Sciences, 194064 Saint-Petersburg, Russia; shalygin.alexey@gmail.com (A.S.); leonid.shuyskiy@gmail.com (L.S.S.); 2 Department of Physiology, Medical College of Wisconsin, Milwaukee, WI 53226, USA; rbohovyk@mcw.edu (R.B.); opalygin@mcw.edu (O.P.); 3 Clement J. Zablocki VA Medical Center, Milwaukee, WI 53295, USA

**Keywords:** podocyte, focal segmental glomerulosclerosis, FSGS, TRPC6, actin cytoskeleton, α-actinin-4

## Abstract

The actin cytoskeleton of podocytes plays a central role in the functioning of the filtration barrier in the kidney. Calcium entry into podocytes via TRPC6 (Transient Receptor Potential Canonical 6) channels leads to actin cytoskeleton rearrangement, thereby affecting the filtration barrier. We hypothesized that there is feedback from the cytoskeleton that modulates the activity of TRPC6 channels. Experiments using scanning ion-conductance microscopy demonstrated a change in migration properties in podocyte cell cultures treated with cytochalasin D, a pharmacological agent that disrupts the actin cytoskeleton. Cell-attached patch-clamp experiments revealed that cytochalasin D increases the activity of TRPC6 channels in CHO (Chinese Hamster Ovary) cells overexpressing the channel and in podocytes from freshly isolated glomeruli. Furthermore, it was previously reported that mutation in ACTN4, which encodes α-actinin-4, causes focal segmental glomerulosclerosis and solidifies the actin network in podocytes. Therefore, we tested whether α-actinin-4 regulates the activity of TRPC6 channels. We found that co-expression of mutant α-actinin-4 K255E with TRPC6 in CHO cells decreases TRPC6 channel activity. Therefore, our data demonstrate a direct interaction between the structure of the actin cytoskeleton and TRPC6 activity.

## 1. Introduction

Podocytes are terminally differentiated epithelial cells whose foot processes wrap the capillaries and form a barrier, which filters the blood in the glomeruli to form primary urine [1]. Podocyte function depends on the actin cytoskeleton and is regulated by multiple proteins and signaling pathways [2]. For many years, angiotensin-converting enzyme inhibitors and angiotensin receptor blockers have been used as the first line of standard nephropathy treatment. A study of the mechanisms of their anti-proteinuric, podocyte-specific effects has demonstrated that calcium-mediated signaling connects angiotensin receptor type 1 with the rearrangement of the cytoskeleton in podocytes [3]. Angiotensin II (Ang II) also plays a critical role in TRPC6-mediated calcium signaling in podocytes [4,5]. TRPC6 (Transient Receptor Potential Canonical 6) calcium channels are essential components of the podocyte slit diaphragm, where they are integrated into a signaling complex that interacts with nephrin, podocin, α-actinin-4, calcineurin, and other proteins [6,7]. Several independent laboratories have identified TRPC6 mutations associated with the autosomal dominant form of focal segmental glomerulosclerosis (FSGS) [8,9,10] and the critical importance of TRPC6 in the maintenance of glomerular filtration. In addition to gain- or loss-of-function mutations that cause FSGS, changes in channel expression may also contribute to the disease [11].

Dysregulation of the actin cytoskeleton leads to foot process retraction, podocyte damage, and proteinuria [12]. TRPC6 activation induces cytoskeleton rearrangements through calcium-dependent signaling in podocytes [13]. Several TRPC6 mutations associated with FSGS result in decreased calmodulin binding and calcium-dependent inactivation of the channels. Increased TRPC6 channel activity changes actin organization from parallel stress fibers to disorganized F-actin structures [14]. TRPC6 channel activation by Ang II also induces cytoskeleton reassembly, decreases the number of actin stress fibers, and promotes a contractile phenotype [15]. Furthermore, it has been shown that TRPC6 inhibition induces cytoskeletal rearrangements of conditionally immortalized mouse podocytes during the differentiation stage [16]. The association between TRPC6 and podocyte actin cytoskeleton rearrangement in diabetic kidney disease has also recently been discussed in a review by Wang et al. [13]. For instance, it was reported that talin1, a focal adhesion molecule, and calpain 1 and 2 are important for maintaining podocyte cytoskeletal stability [17,18] and the TRPC6-mediated Ca^2+^-dependent calcineurin signal transduction pathway [19]. TRPC6 also binds to and activates the actin regulatory proteins caldesmon and calpain 1 and 2, proteases that control the podocyte cytoskeleton, cell adhesion, and motility via cleavage of paxillin and talin [20].

α-actinin-4, encoded by the *ACTN4* gene, cross-links filamentous actin into thick bundles with defined spacing and is also regulated by calcium signaling. The binding efficacy of α-actinin-4 to actin is modified by the calmodulin-like domain of α-actinin-4 [21]. Moreover, α-actinin-4 interaction with the cytoskeleton depends on phosphorylation [22,23], which is diminished in cells with the FSGS-causing mutation ACTN4 K255E [24]. ACTN4 K255E is a gain-of-function mutation which increases actin binding and eliminates sensitivity to tension [25]. Binding of mutant α-actinin-4 enhances cytoskeleton stiffness, contractile force, and recovery time after stretch [26,27], and decreases cell spreading [28]. Many cases of FSGS are caused by disruption of TRPC6 calcium entry or the cytoskeleton’s rearrangements [29]. Since mutations in TRPC6 are known to cause cytoskeleton rearrangement, we propose there is a relationship between cytoskeleton dynamics and TRPC6 channel activity. The goal of this study was to study how cytoskeleton reorganization influences TRPC6 channel activity.

## 2. Results

### 2.1. Disruption of the Actin Cytoskeleton with Cytochalasin D Affects Filopodial Extension and Cell Migration

**Figure 1:**
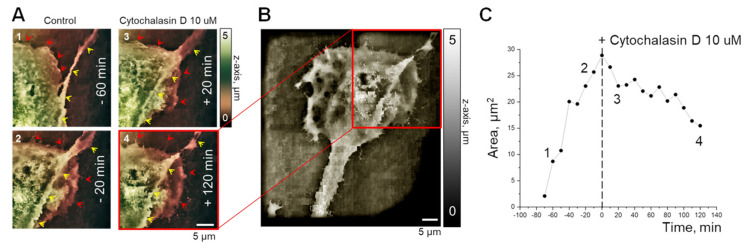
Scanning Ion Conductance Microscopy (SICM) analysis of human podocyte morphology in response to cytochalasin D treatment. (**A**) Representative scans of podocyte lamellipodia (red arrows) and filopodia (yellow arrows) at different time points (60 and 20 min before (1–2) and 20 and 120 min after (3–4) cytochalasin D (10 µm) application; scan area 30 µM × 30 µM). (**B**) Expanded topographical map showing the complete podocyte from **a** after the application of cytochalasin D (scan area 60 µM × 60 µM). (**C**) Timeline of changes to the podocyte foot process area during normal migration and after cytochalasin D application. Numbers 1–4 represent time points shown in (**A**). As seen in this summary, cytochalasin D completely stopped the migration of the podocyte filopodium.

To test the remodeling effects of the actin cytoskeleton on podocyte morphology, we employed a novel scanning ion-conductance microscopy (SICM) approach. SICM generates electron microscopy resolution images of live cells in physiologically relevant solutions over time. Experiments using SICM demonstrate that disrupting the actin cytoskeleton integrity by application of cytochalasin D prevents changes in podocyte foot process and migration area. Cytochalasin D is a cell-permeable fungal toxin that binds to the barbed end of actin filament, inhibiting both the association and dissociation of actin subunits. Actin dynamics allow cells to change shape in response to specific stimuli. The actin cytoskeleton form highly organized dynamic structures, such as filopodia and lamellipodia. Lamellipodia (red arrows, Figure 1a) and filopodia (yellow arrows, Figure 1a) are actin-based protrusions particularly relevant to cell motility. Figure 1a shows a detailed 3D topographical image of a podocyte foot process scanned in real-time to observe the changes during the physiological migration process and under treatment with cytochalasin D. To estimate the activity of cell edge migration, we calculated the scanned area at different time points during normal physiological conditions and after the application of cytochalasin D (Figure 1). A stable increase in the cell area was calculated in the scanned cell fragment (Figure 1a,c (images 1 and 2); time frame from −70 to 0 min). The application of cytochalasin D resulted in the retraction of the podocyte migratory cell edge, thereby decreasing the calculated area of the cell edge (Figure 1a,c (images 3 and 4); time frame from 0 to +120 min). A full cell scan (Figure 1b) requires a lack of sample movement for its long duration (~40 min), therefore the full image was made after the disruption of the actin cytoskeleton and prevention of cell migration by cytochalasin D treatment.

### 2.2. Acute Disruption of Actin Microfilaments with Cytochalasin D Markedly Increases TRPC6 Activity (NP_o_) in CHO Cells and Rat Podocytes

**Figure 2:**
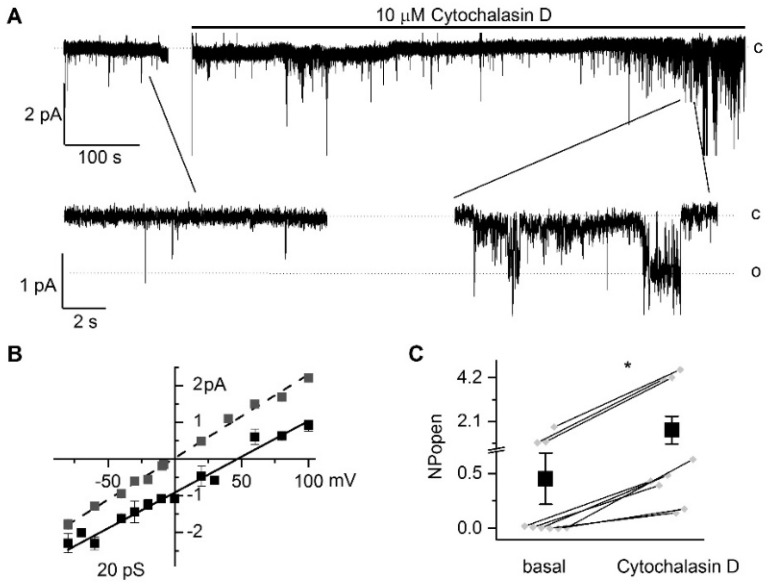
Effect of cytochalasin D on TRPC6 (Transient Receptor Potential Canonical 6) channels overexpressed in CHO (Chinese Hamster Ovary) cells. (**A**) Representative trace of TRPC6 channel activity before and after application of 10 µM cytochalasin D at a holding potential of –40 mV. Expanded fragments are shown below. Closed (c) and open (o) states are indicated. (**B**) Current-voltage relationship of overexpressed TRPC6 with estimated conductance 20 ± 1 pS in cell-attached mode (black squares) and subsequent excision to inside-out mode (grey squares). (**C**) Summary graph demonstrating the effect of the application of 10 µM cytochalasin D on TRPC6 channel activity (NP_o_). Wilcoxon nonparametric test for paired samples; * = *p* < 0.05.

To study the regulation of TRPC6 by disruption of actin cytoskeleton dynamics, we tested the effects of cytochalasin D on TRPC6 channel activity. Several members of the TRPC family, including TRPC3, TRPC5, and TRPC6, have been implicated in the pathogenesis of proteinuric kidney diseases [30,31,32]. Therefore, to test the effect of the actin cytoskeleton on TRPC6 channels, we heterologously overexpressed TRPC6 in CHO (Chinese Hamster Ovary) cells. The application of 10 of µM cytochalasin D to a bath solution of cell-attached patches induced activation of single channels (Figure 2a) with a current-voltage relationship typical for TRPC6 (Figure 2b). Application of cytochalasin D did not affect conductance of the recorded channels (18 ± 1 pS (*n* = 3) and 20 ± 1 pS (*n* = 5) before and after cytochalasin D application, respectively). The positive reversal potential of the current-voltage relationship reflects the plasma membrane potential of the cell. After excision to inside-out configuration, the reversal potential was shifted to 0 mV, and the conductance increased to 23 ± 1 pS (*n* = 6). Control cells transfected with GFP alone lacked TRPC6 activity before and after cytochalasin D application. Basal activity (NP_o_) of overexpressed TRPC6 channels was increased after the application of cytochalasin D (Figure 2c). In experiments with no basal activity, 10 µM of cytochalasin D also activated TRPC6 channels but with less activity, which most likely represents a smaller number of active channels. On average, NP_o_ increased from 0.45 ± 0.23 to 1.69 ± 0.66 (Figure 2c).

**Figure 3:**
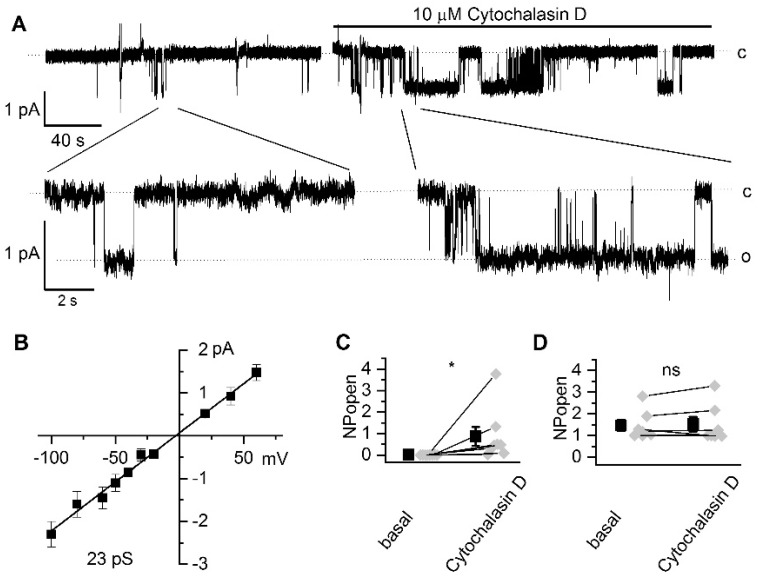
Application of cytochalasin D to podocytes from freshly isolated glomeruli induces TRPC6 channel activation. (**A**) Representative example of basal channel activity before and after 10 µM cytochalasin D application at –40 mV holding potential. Expanded fragments are shown below. Open and closed channel states are indicated with (o) and (c), respectively. (**B**) Current-voltage relationship of recorded channels. (**C**) Summary graph of the endogenous silent TRPC6 activity induced by 10 µM cytochalasin D in podocytes. (**D**) Summary graph demonstrating the effect of the application of 10 µM cytochalasin D on constitutive endogenous TRPC6-like channel activity (NP_o_) in freshly isolated glomeruli. Wilcoxon nonparametric test for paired samples; * = *p* < 0.05.

In our next experiments, we tested the effect of inhibiting actin cytoskeleton dynamics on endogenous TRPC channels. We performed cell-attached patch-clamp experiments on podocytes from freshly isolated, decapsulated glomeruli. In cell-attached experiments, the addition of 10 µM of cytochalasin D to the bath solution induced activation of currents (Figure 3a) with current-voltage relationships and conductance of 23 ± 1 pS, typical for TRPC6 channels (Figure 3b). The increase in calcium entry is caused by the activation of silent TRPC6 channels and is not associated with changes in their conductivity or other biophysical properties (Figure 3). The NP_o_ of silent channels increased from 0.00 to 0.87 ± 0.44 (*n* = 8; Figure 3c). In contrast to experiments with CHO cells, basal TRPC6 activity was not significantly increased after cytochalasin D application (1.46 ± 0.25 vs 1.52 ± 0.34, *n* = 7, Figure 3d).

**Figure 4:**
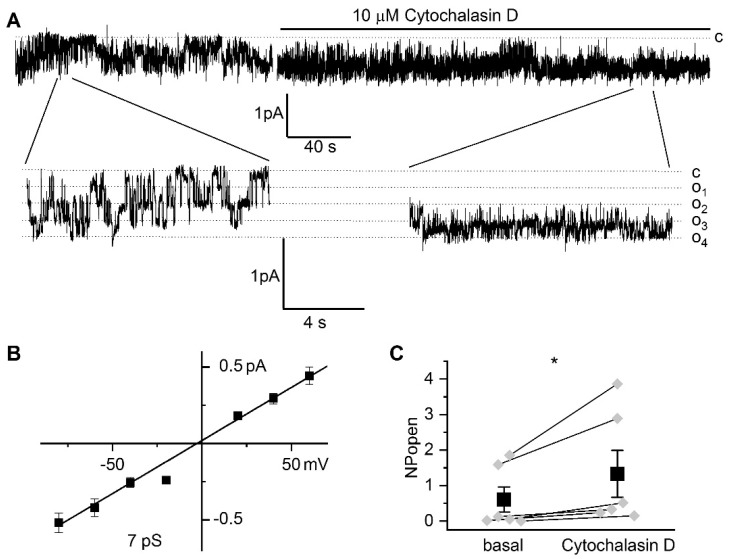
Application of cytochalasin D to podocytes induces activation of a native channel with 7 pS conductance. (**A**) Representative trace of basal channel activity before and after 10 µM cytochalasin D at a holding potential of –40 mV. Expanded fragments are shown below. Closed (c) and open (o_i_) states are indicated. (**B**) Current-voltage relationship of channels with estimated conductance 7.0 ± 0.2 pS. (**C**) Summary graph of the effect of 10 µM cytochalasin D on 7 pS channel activity (NP_o_). Wilcoxon nonparametric test for paired samples; * = *p* < 0.05 level.

Interestingly, in addition to TRPC6-like channels (*n* = 15), we observed activity of another endogenous channel (Figure 4a) with 7.0 ± 0.1 pS conductance (*n* = 6, Figure 4b); in 3 experiments, both types of channels were observed, while in 8 attempts there was no channel activity. Cytochalasin D increased the activity of 7 pS channels with NP_o_ augmented from 0.61 ± 0.35 to 1.33 ± 0.66 (Figure 4c). Additional studies are required to determine the identity of this channel.

### 2.3. ACTN4 K255E Reduces TRPC6 Activity (NP_o_) and Conductance

**Figure 5:**
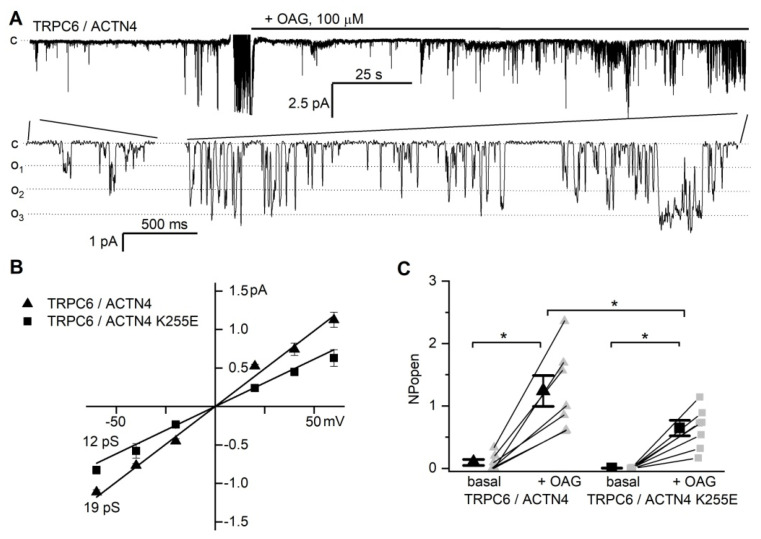
Activities of TRPC6 channels in response to OAG in CHO cells co-transfected with wild type ACTN4 (α-actinin-4) or ACTN4 K255E. (**A**) Typical trace of OAG-induced TRPC6 activity in CHO cells co-transfected with ACTN4; (c) and (o_i_) denote closed and open states of the channel, respectively; a full recording (upper row) and fragments of a recording at a larger scale are shown. The recording was obtained at holding potential –60 mV. (**B**) Summarized current-voltage relationship curve of TRPC6 currents of CHO cells co-transfected with ACTN4 wild-type (triangle) with estimated conductance 19±1 pS and ACTN4 K255E mutant (square) with estimated conductance 12 ± 2 pS. (**C**) Summary graphs of the NP_o_ of the TRPC6 channels recorded in CHO cells co-transfected with TRPC6 and ACTN4 or ACTN4 K255E before and after OAG (100 µM) stimulation; * denotes statistical significance (*p* < 0.05).

Since the actin-binding protein α-actinin-4 plays an essential role in podocyte function and mutations in the *ACTN4* gene, which encodes this protein, are associated with FSGS, we interrogated whether α-actinin-4 can modulate TRPC6 channel activity. The FSGS-causing ACTN4 mutation K255E increases podocyte cytoskeleton stiffness [26,27,33]; thus, we hypothesized that it could affect TRPC6 activity. To test this, we compared the activity of TRPC6 channels in CHO cells co-transfected with wild-type (wt) ACTN4 or the K255E mutant. Moderate basal activity of TRPC6 channels was observed using cell-attached experiments in CHO cells co-transfected with TRPC6 and wild type ACTN4 (TRPC6/ACTN4), whereas no basal activity was observed in CHO cells with TRPC6 and ACTN4 K255E (TRPC6/ACTN4 K255E) (Figure 5). To further explore the effects of α-actinin-4 on TRPC6 activity, we applied OAG, a membrane-permeant analog of diacylglycerol, which is an agonist of TRPC channels. In cell-attached experiments, the addition of 100 µM of OAG into the bath solution induced TRPC6 channel activity in both TRPC6/ACTN4 and TRPC6/ACTN4 K255E cells (Figure 5). Current-voltage relationships demonstrated that the conductance of TRPC6 channels in TRPC6/ACTN4 cells was 19 ± 1 pS, similar to the properties of TRPC6 channels in podocytes or CHO cells transfected with TRPC6 only (Figure 5b). Interestingly, TRPC6 channels in TRPC6/ACTN4 K255E cells exhibited a significantly lower conductance of around 12 ± 2 pS [34]. Estimation of OAG-induced TRPC6 activity in TRPC6/ACTN4 cells showed that NP_o_ increased from 0.09 ± 0.05 to 1.24 ± 0.23 (*n* = 7), comparable with OAG-induced TRPC6 activity CHO cells transfected with TRPC6 alone (from 0.05 ± 0.04 to 1.40 ± 0.33 (n = 8)). In TRPC6/ACTN4 K255E cells, channel activity upon OAG stimulation increased from 0.00 to 0.65 ± 0.12 (*n* = 7). Therefore, the ACTN4 K255E mutation reduces both TRPC6 channel conductance and current activity.

**Figure 6:**
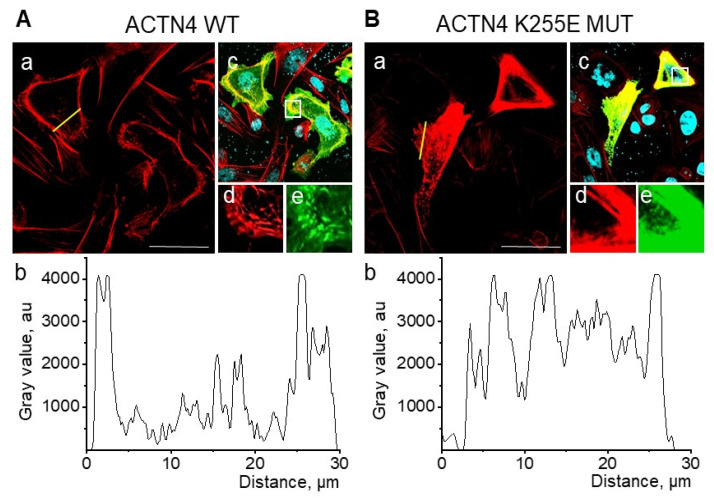
Effect of ACTN4 K255E mutant on actin cytoskeleton arrangement. CHO cells were transiently transfected with plasmids encoding wild type (**A**) or mutant K255E ACTN4 (**B**). Scale bar is 50 μm. a—rhodamine-phalloidine emission (red); b—histogram of relative fluorescence across a region of interest (yellow line); c—merged image of rhodamine-phalloidine (red), GFP-labeled α-actinin-4 (green) and Hoechst-33342 (nuclei, blue) emissions. Panels (d) and (e) are zoomed areas from panel (c) (marked by white square), for rhodamine-phalloidine and GFP-labeled α-actinin-4, respectively.

To confirm that actin cytoskeleton rearrangements by ACTN4 K255E overexpression did in fact lead to the observed reduction of TRPC6 single-channel activity, we analyzed actin cytoskeleton arrangement using confocal scanning microscopy. Representative images of actin cytoskeleton staining by rhodamine-phalloidine in CHO cells transfected with ACTN4 wt and ACTN4 K255E mutant are presented in Figure 6a,b. The structure of the actin cytoskeleton in cells transfected with ACTN4 K255E mutant was altered compared to controls transfected with wild-type ACTN4. ACTN4 K255E expression resulted in enhanced actin bundle thickness (Figure 6b panels a, d, e) and abnormal actin cytoskeleton distribution (Figure 6b panels a and b). These results are consistent with previously published data showing that the K255E mutation in ACTN4 alters the actin cytoskeleton structure and impacts different cell processes, including migration, contractility, and tensile response [24,27,33].

## 3. Discussion

Podocytes are exposed to repetitive stretch and shear stress, and their filtration function requires the actin cytoskeleton [35]. It was previously shown that actin cytoskeleton rearrangements are induced by activation of small GTPases RhoA and Rac1, which modulate calcium signaling after TRPC channel activation [36]. It is also well established that FSGS is associated with mutations in TRPC6 channels and actin cytoskeleton associated proteins [29]. However, the ability of the actin cytoskeleton to influence TRPC6 activity has not previously been investigated. Our study demonstrates that TRPC6 channel activity is impacted by inhibition of the actin cytoskeleton in podocytes. Our results are in agreement with the observation that cytochalasin D increases stretch-evoked whole-cell currents in an immortalized human podocyte cell line [37]. TRPC6 channels are not inherently sensitive to membrane stretch, unlike the classic stretch sensitive channel Piezo1 [38,39]; however, their mechanosensitivity properties may be linked to intracellular tethers. Stretch evoked activation of TRPC6 is modulated by podocin [37] or cytoskeleton components [31]. Our experiments disrupting the actin cytoskeleton suggest that TRPC6 association with actin networks may also influence channel activity.

In podocytes from freshly isolated glomeruli, we observed two modes of TRPC6 channel activity; active channels that were not further modified by cytochalasin D application and silent channels that were activated by cytochalasin D. Our results demonstrate that application of cytochalasin D to podocytes induces the activation of silent channels only and is not able to change the properties of highly active channels. Furthermore, it appears that the number of active channels does not change, at least during acute treatment with cytochalasin D.

In addition to TRPC6 channels, we observed additional channels with a smaller conductance, that could also be activated by the application of cytochalasin D. Channels with similar properties have previously been described in podocytes [34]. Potentially, they could be composed of TRPC heteromeres with lower permeability [40,41,42]; however, it is also possible that other types of endogenous channels are activated in response to inhibition of actin cytoskeleton dynamics. Additional studies are required to reveal the identity of these channels.

Both the application of cytochalasin D and the expression of the α-actinin-4 K255E mutant resulted in changes to cell morphology (Figure 1 and Figure 6); however, their effects on TRPC6 activity were different. While in both cases we demonstrated that TRPC6 activity is under the control of the cytoskeleton, the different mechanisms of cytoskeletal perturbation cause disparate effects on channel activity. Thus, further experiments are needed to more fully understand the underlying dynamics by which the actin cytoskeleton influences TRPC6 behavior. It was reported that TRPC6 mechanosensitivity is linked to cytoskeleton rearrangement [37], while TRPC6 regulation by DAG after receptor activation is controlled by other pathways [43]. There are likely multiple factors at play that need additional investigation in order to understand these phenomena.

α-actinin-4, encoded by ACTN4, is an essential protein controlling podocyte structure and function. Upregulation of α-actinin-4 is necessary for the differentiation of stem cells to podocytes [44,45]. Expression of α-actinin-4 is decreased in a model of diabetic nephropathy as well as in FSGS caused by the ACTN4 K255E mutation [46,47]. The ACTN4 K255E mutation also reduces foot process-like peripheral projections in a conditionally immortalized podocyte cell line [33], similar to the disturbances in dendritic spine dynamics of neurons lacking α-actinin-4 [48]. In addition to podocytes, α-actinin-4 has been established as a critical mechanoresponsive protein in several other tissues [49,50]. Mutant ACTN4 K255E has increased binding affinity for actin [21] and slower recovery from tension [25,35]. α-actinin-4 binding to actin is regulated and reduced by calcium or phosphorylation at tyrosines 4 and 31 [28,51] or increased by phosphorylation at position S159 [23]. Mutant ACTN4 K255E is insensitive to calcium, while phosphorylation at tyrosines 4 and 31 decreases its actin-binding [24]. There is limited information about the relationship between α-actinin-4 and ion channels. Our results demonstrate a novel regulatory effect of mutant α-actinin-4 on TRPC6 channel function. In cells expressing ACTN4 K255E, we observed reduced TRPC6 channel activity (both basal and OAG-activated), while the number of channels was unaltered. α-actinin-4 was also reported to be associated with acid-sensing ion channel ASIC1a. Similarly, it was shown that co-expression of α-actinin-4 with ASIC1a did not affect cell surface expression. In contrast, α-actinin-4 altered ASIC1a current density, pH sensitivity, desensitization rate, and recovery from desensitization [52]. These results suggest an additional potential mechanistic role for ACTN4 K255E to influence podocyte calcium entry through TRPC6 in the pathogenesis of FSGS.

In summary, our data demonstrate that the dynamic rearrangements of the cytoskeleton can affect calcium entry through TRPC6 channels, which in turn can influence the filtering properties of podocytes.

## 4. Materials and Methods

### 4.1. Cells

CHO cells were cultured in a DMEM/F12 medium supplemented with 10% heat-inactivated FBS and 80 μg/mL of gentamicin. For experiments measuring TRPC6 activity, CHO cells were co-transfected using a PEI transfection reagent (Polysciences Inc., Warrington, FL, USA) with 0.5 µg TRPC6 and 0.25 µg GFP, 24–48 h before electrophysiological analysis. For control experiments, CHO cells were transfected with 0.7 µg GFP. For experiments with α-actinin-4, the cells were co-transfected with TRPC6 tagged with GFP and wild-type or mutant ACTN4 K255E tagged with GFP [27]. For control experiments with α-actinin-4, CHO cells were transfected with TRPC6 tagged with GFP. The weight ratio of plasmid DNAs was: TRPC6—0.7 µg; ACTN4—0.7 µg. For cytoskeleton analysis, CHO cells were transiently transfected by ACTN4 wt, or ACTN4 K255E mutant (0.7 µg of each DNA, respectively), 24 h before experiments.

The immortalized human podocyte cell line AB 8/13 was kindly provided by M. Saleem and has been described previously [53,54]. Cells were cultured in an RPMI-1640 medium supplemented with 10% heat-inactivated FBS and insulin-transferrin-selenium supplement. Podocytes proliferate abundantly at 33 °C, and after thermo-switching to 37 °C (for 10 days), cells become mature podocytes and express key structural proteins such as nephrin and podocin.

### 4.2. Animals

Male Wistar 9–14-week-old rats were used for experiments. The glomerular isolation protocol has been described previously [34]. Briefly, in anesthetized rats, the abdominal cavity was cut, and the kidneys were excised and placed in a cold saline solution. All actions were carried out in accordance with the rules of the local ethical committee. After isolation of the kidneys, the renal capsule was removed, and the kidney cortex was isolated with a thickness of 1–2 mm. The cortex of the kidney was crushed into small chunks and passed through a special mesh grid filters with numbers of 80, 120, and 200 (pore sizes of 150, 106, and 73.7 µm, respectively). After glomerular isolation, the podocytes are visible under a light microscope (40×). They have a round or oval shape and are located on the surface of the glomerular capillary loops.

### 4.3. Electrophysiological Analysis

For cell-attached patch-clamp analysis, solutions were (in mM): bath—NaCl 130, CaCl_2_ 1, HEPES 10, MgCl_2_ 2, Glucose 10, pH 7.4 (in experiments with ACTN4—130 mM NaCl was replaced by 140 mM KCl, 5 mM NaCl); pipette—NaCl 126, CaCl_2_ 1.5, HEPES 10, Glucose 10, TEACL 10. A number of blockers were added to the pipette solution, specifically 10 nM of iberiotoxin, 0.1 mM of DIDS, and 10 µM of nicardipine. The inhibitors prevented activation of non-TRPC cationic channels from interfering with patch-clamp recordings. Voltage is amplifier command potential. Positive current flows from the pipette into the cell. Additional filtering at 300 Hz was applied before analysis. Cytochalsin D and OAG was from Sigma-Aldrich (St. Louis, MO, USA).

Single-channel unitary current (i) was determined from the best-fit Gaussian distribution of amplitude histograms. The activity was analyzed as NP_o_ = I/i, where I is the mean total current in a patch and i is unitary current at this voltage.

### 4.4. Imaging of the Cytoskeleton

Fixation and staining of the transfected CHO cells were performed according to a standard protocol [55]. Cells were passaged onto coverslips (12 mm × 12 mm), washed with PBS the next day after transient transfection (by wild type ACTN4 or ACTN4 K255E mutant), and then fixed with 3.7% formaldehyde in PBS for 10 min at room temperature. Then, the cells were perforated with 0.1% Triton X-100 (5 min, room temperature) in PBS and incubated with 2 μM rhodamine-phalloidin solution (Sigma-Aldrich) for 15 min at 37 °C. Nuclei were stained with a Hoechst-33342 dye (5 μg/mL, 5 min incubation, room temperature) and mounted onto a slide using Vectashield medium (Vector Laboratories, Inc., Burlingame, CA, USA). The addition of each reagent (pre-dissolved in PBS) was followed by washing with PBS. Imaging was carried out using Olympus FV3000, × 63 lens, digital zoom. Lasers with excitation wavelengths of 405 nm (Hoechst-33342, emission maximum at 461 nm), 488 nm (GFP, emission maximum at 509 nm), and 561 nm (rhodamine-phalloidin, emission maximum at 565 nm) were used. Image analysis and processing were performed using open-source software ImageJ v1.53c (National Institutes of Health, USA, http://imagej.nih.gov/ij/).

### 4.5. Scanning Ion Conductance Microscopy (SICM) Analysis

To perform SICM imaging, human podocytes were attached to poly-l-lysine glass surface and placed into the cell chamber filled with PSS solution. Samples were manually positioned in the *x*–*y* direction under the inverted optical microscope, Nikon TE2000-U (Nikon Instruments, Tokyo, Japan). For the hopping probe, SICM imaging borosilicate glass nanopipettes with a resistance of approximately 100 MΩ, which corresponds to an estimated tip diameter of around 120 nm, were used as described previously [56,57]. The nanopipettes were filled with the same PSS solution used for the bath and were positioned in the z-direction with a piezoelectric actuator. The ion current flowing through the nanopipettes was measured with an Axopatch 700B patch-clamp amplifier (Molecular Devices, San Jose, CA, USA) in voltage-clamp mode and monitored by the custom-modified universal controller (ICAPPIC Ltd., London, UK), which simultaneously controlled sample and pipette positioning [58].

### 4.6. Statistical Analysis

Results are presented as a mean ± standard error of the mean. Unpaired Student *t*-test results were calculated using Origin software (Microcal Software, Northampton, MA, USA). Differences with *p* < 0.05 were considered statistically significant.

## Data Availability

The data presented in this study are available upon reasonable request from the corresponding author.
